# Progress in understanding and treating idiopathic pulmonary fibrosis: recent insights and emerging therapies

**DOI:** 10.3389/fphar.2023.1205948

**Published:** 2023-08-07

**Authors:** Hehua Guo, Jiazheng Sun, Siyu Zhang, Yalan Nie, Sirui Zhou, Yulan Zeng

**Affiliations:** Department of Respiratory Medicine, Liyuan Hospital, Tongji Medical College, Huazhong University of Science and Technology, Wuhan, China

**Keywords:** idiopathic pulmonary fibrosis, etiology, pathogenesis, treatment, progress

## Abstract

Idiopathic pulmonary fibrosis (IPF) is a long-lasting, continuously advancing, and irrevocable interstitial lung disorder with an obscure origin and inadequately comprehended pathological mechanisms. Despite the intricate and uncharted causes and pathways of IPF, the scholarly consensus upholds that the transformation of fibroblasts into myofibroblasts—instigated by injury to the alveolar epithelial cells—and the disproportionate accumulation of extracellular matrix (ECM) components, such as collagen, are integral to IPF’s progression. The introduction of two novel anti-fibrotic medications, pirfenidone and nintedanib, have exhibited efficacy in decelerating the ongoing degradation of lung function, lessening hospitalization risk, and postponing exacerbations among IPF patients. Nonetheless, these pharmacological interventions do not present a definitive solution to IPF, positioning lung transplantation as the solitary potential curative measure in contemporary medical practice. A host of innovative therapeutic strategies are presently under rigorous scrutiny. This comprehensive review encapsulates the recent advancements in IPF research, spanning from diagnosis and etiology to pathological mechanisms, and introduces a discussion on nascent therapeutic methodologies currently in the pipeline.

## 1 Introduction

Idiopathic pulmonary fibrosis (IPF) is a pervasive chronic pulmonary ailment marked by irreversible lung function loss and structural disfigurement attributable to an overproduction of extracellular matrix deposition (Mei et al., 2021), compounded by progressive scarring of lung tissue and interstitial lung disease (Qian et al., 2021). The disease is predominantly observed in middle-aged to elderly men, and its global prevalence is estimated to exceed 3 million people, with an annual incidence rate between 2 and 9 per 100,000 individuals and an escalating trend (He et al., 2021a). The mean survival time post-diagnosis is a mere 3–5 years (Lambert et al., 2021), and clinically, IPF is associated with dyspnea, a relentless decline in lung function, a dismal prognosis, and disease trajectories that span from gradual deterioration to swift collapse, culminating in mortality due to respiratory failure (Zhang et al., 2022).

The exact origins and progression mechanisms of IPF remain ambiguous, with aging recognized as the most considerable risk factor (Roque et al., 2020a). Additional contributing factors include genetics, environmental exposure, smoking habits, viral infections, gastroesophageal reflux disease, and fibrogenesis, all playing roles in IPF manifestation (Mostafaei et al., 2021). Therapeutic interventions are currently limited, with only two FDA-endorsed pharmaceuticals in the United States, namely, pirfenidone and nintedanib (Shenderov et al., 2021). While both drugs mitigate the pace of IPF progression and lung function degradation, they fall short of reversing the lung damage inflicted by the disease (Ma et al., 2022). Pirfenidone, a small molecule pyridine, possesses anti-fibrotic, anti-inflammatory, and antioxidant properties (Aimo et al., 2020), acting to inhibit fibrotic processes via the suppression of the cytokine TGF-β (Petnak et al., 2021). Nintedanib is a small molecule tyrosine kinase inhibitor that targets receptors, leading to a broad inhibition of downstream signaling pathways in fibroblasts and myofibroblasts (Wind et al., 2019). Both drugs function to curb fibroblast proliferation, obstruct collagen production, and diminish fibrogenic mediator production (Tanaka et al., 2022).

Presently, lung transplantation stands as the sole clinically validated effective treatment strategy (Han et al., 2022), yet it is encumbered by high costs, a dearth of compatible donors, and post-operative rejection risks, significantly constraining its clinical utility. The principal objective of IPF management is to alleviate symptoms, enhance patient health and quality of life, and maintain lung function as a means to extend survival (Olson et al., 2018). The importance of a profound comprehension of IPF’s etiology and pathogenesis for early diagnosis and efficacious treatment has been underscored through long-term clinical practice. The past two decades have witnessed significant strides in basic and clinical research on IPF, both domestically and internationally. This review is intended to compile and present the advancements in understanding the etiology, pathogenesis, and therapeutic approaches of idiopathic pulmonary fibrosis.

## 2 Etiology

### 2.1 Genetic

Genetic constituents serve as fundamental drivers in the initiation and evolution of IPF (Ballester et al., 2019). A growing body of evidence indicates that susceptibility to IPF is linked to a complex interplay of genetic variations and alterations in transcriptional activity (Wolters et al., 2018). Mutations in the mucin 5B gene have emerged as one of the most influential risk factors for IPF (Wang et al., 2022). A pivotal study unveiled a prevalent single nucleotide polymorphism in the promoter domain of the MUC5B gene on chromosome 11 (Stainer et al., 2021), predominantly associated with pulmonary fibrosis (Biondini et al., 2021). Telomeres, non-coding, repetitive nucleotide sequences situated at chromosome extremities, shield chromosomes from progressive attrition during typical cellular replication (Drakopanagiotakis et al., 2018). Rare aberrations in genes associated with telomere homeostasis have been strongly implicated in pulmonary fibrosis (Wells et al., 2018). It has been observed that nearly all sporadic IPF patients present shortened telomeres in alveolar epithelial cells (Sgalla et al., 2018). Furthermore, both acute and chronic fibrotic lung diseases transpire in patients with mutations in pulmonary surfactant apolipoprotein and lipid transporter (Plantier et al., 2018), insinuating a significant role of surfactant composition or metabolic alterations in IPF.

### 2.2 Environmental exposure

Frequently, particulate matter, fibers, and dust constitute the primary environmental contributors to IPF onset (Phan et al., 2021). A noteworthy surge in IPF incidence is observable among individuals with exposure to animal dust, chemical fumes, metal dust (including lead and steel), and other pollutants (Walters, 2020). These contaminants are identified as precipitators of oxidative stress, epithelial damage, and airway inflammation (Sack and Raghu, 2019). Studies suggest that air pollution may incite epigenetic modifications in the lung, enhancing pathogenicity in synergy with other antigens (Park et al., 2021), and potentially initiating or fostering disruptions in alveolar damage and repair mechanisms. Post-mortem examinations of IPF patients have detected inorganic particles in lung lymph nodes (Collins et al., 2018), further corroborating the environmental exposure etiology.

### 2.3 Lung microbiota

Recent investigations underscore the crucial role of microbiota in inciting and exacerbating pulmonary fibrosis in animal models, thereby elucidating the association between microbiota and pulmonary fibrosis (Spagnolo et al., 2019). Contemporary characterizations of the respiratory microbiota in IPF suggest that an escalated bacterial burden and the presence of specific organisms may be instrumental in disease onset (Molyneaux et al., 2014). Some viruses have also been implicated in initiating, promoting, or intensifying IPF (Phan et al., 2021). Bacteria and viruses can inflict damage on airway epithelial cells directly or indirectly through the activation of immune responses to infection (Sack and Raghu, 2019). Evidence indicates that extracellular vesicles generated by certain Gram-negative bacteria, resulting from lung microbiota dysregulation, instigate the expression of pro-inflammatory and pro-fibrotic genes across a variety of cell types. Additionally, IL-17B and TNF-a secreted by these extracellular vesicles interact to construct an inflammatory network system conducive to pulmonary fibrosis (Yang et al., 2019). Research conducted by David and colleagues unveiled a significant correlation between the lung microbiota burden and IPF progression (O'Dwyer et al., 2019). Furthermore, it has been reported that the mortality risk in IPF escalates with the increasing bacterial burden in the lung (Molyneaux et al., 2014).

### 2.4 Smoking

Recognized as a principal risk factor for chronic respiratory diseases such as COPD (Aghapour et al., 2018), smoking also has a significant role in precipitating pulmonary fibrosis. A potent correlation exists between smoking and IPF, which amplifies with the escalation in dosage, especially evident in habitual smokers or those who have smoked over extended periods (Bellou et al., 2021). Cigarette smoke can inflict damage on all lung cell types, particularly alveolar epithelial cells, giving rise to diffuse infiltration and parenchymal fibrosis (Kumar et al., 2018). One investigation indicated that IPF patients with a long-term smoking history exhibited lower overall cell density, albeit higher alveolar cell density and more severe damage (Zhang et al., 2021). One study established that cigarette smoking correlates with an augmented risk of IPF, with ever-smokers facing a 60% elevated risk (Bae et al., 2022).

### 2.5 Gastroesophageal reflux disease

The pronounced incidence of gastroesophageal reflux disease (GERD) in IPF suggests a pathogenic role for microaspiration attributable to GERD (Bedard Methot et al., 2019). Chronic microaspiration stemming from GERD is deemed a likely precursor to IPF (King and Nathan, 2017). For individuals predisposed to IPF, chronic microaspiration induced by gastric reflux can inflict enduring, recurrent damage to lung tissue, increasing lung epithelial cell permeability, continually stimulating pulmonary fibrosis proliferation, and eventually contributing to the manifestation of pulmonary fibrosis (Ghisa et al., 2019). Several cell biology experiments and preclinical studies indicate that gastric reflux constituents, such as acid and proteases, can elicit adverse effects such as immune response stimulation, enhanced cell membrane permeability, severe airway inflammation, and lung tissue structure alteration (Nelkine et al., 2020). Evidence supporting this hypothesis emerges from descriptive studies conducted in both experimental animal models and humans (Chen et al., 2019; Reynolds et al., 2023).

### 2.6 Aging

The mechanism by which aging leads to pulmonary fibrosis remains unclear. Cellular senescence can disrupt various cellular biological activities in the body, manifesting as telomere attrition, DNA damage, and mitochondrial dysfunction (Merkt et al., 2020). Research indicates that cellular senescence induced by telomere degradation primarily afflicts alveolar epithelial type II cells, which is intricately linked to IPF pathogenesis (Stuart et al., 2014; Selman et al., 2019). Repeated microdamage to senescent epithelial cells in genetically susceptible individuals can trigger abnormal fibroblast activation, culminating in ECM accumulation and fibrosis (Pardo and Selman, 2016). Multiple murine models of pulmonary fibrosis display evidence of various cellular senescence markers, including heightened aging-associated β-galactosidase in lysosomes, an increased BCL-2/Bax ratio of apoptosis-involved proteins in mitochondria, and amplified DNA damage in the nucleus (Schafer et al., 2017; Blokland et al., 2020), all coupled with robust pro-fibrotic effects, such as TGF-β (Rana et al., 2020). Aging can impede stem cell turnover functionality, obstructing the repair and regeneration of alveolar epithelial cells in damaged lungs (Mei et al., 2021). Aging has been implicated in promoting fibrosis by thwarting blood vessel regeneration (Chen et al., 2021). IPF prevalence and incidence continue to rise in individuals over 65 years and remain exceedingly rare in those under 50 years old (Raghu et al., 2018), reinforcing the classification of IPF as an aging-related disease.

## 3 Pathogenesis

The pathogenesis of IPF is multifaceted and, as of yet, not wholly comprehended. Nevertheless, several pivotal factors have been pinpointed as significant contributors to the disease’s inception and evolution. Herein is a synopsis of our current understanding of IPF pathogenesis.

### 3.1 Transforming growth factor (TGF)

TGF-β is considered a central component among the diverse factors contributing to pulmonary fibrosis development (Chanda et al., 2019). Released in response to epithelial cell injury, TGF-β acts as a key upstream pro-fibrotic growth factor propelling the disease’s pathophysiology (Shimbori et al., 2019; He et al., 2021b). As a multifunctional cytokine, TGF-β fosters pulmonary fibrosis through a range of mechanisms (Nolte and Margadant, 2020). Primarily, it incites the proliferation and differentiation of epithelial cells and fibroblasts (Ghavami et al., 2018), spurs myofibroblasts to generate the extracellular matrix, catalyzes epithelial-mesenchymal transition, expedites epithelial apoptosis and cell migration, and provokes the production of connective tissue growth factor among other mediators (Phan et al., 2021). TGF-β not only choreographs the congregation of fibroblasts at injury sites but also facilitates their metamorphosis into myofibroblasts (Ye and Hu, 2021). Moreover, TGF-β distinguishes itself as the most efficacious extracellular matrix production stimulant and is considered the strongest chemoattractant for immune cells, including monocytes and macrophages (Kinoshita and Goto, 2019).

### 3.2 Insulin-like growth factor (IGF)

The insulin-like growth factor has a significant role in pulmonary fibrosis progression (Kheirollahi et al., 2022). Composed of 70 amino acids, IGF-1 chiefly mediates a range of biological functions, including cell division, differentiation, apoptosis, and metabolism (Jensen-Cody and Potthoff, 2021; Jiang et al., 2023). IGF-1 is postulated to facilitate fibroblast proliferation, migration, and differentiation, augmenting the ability of fibroblasts to synthesize fibronectin and collagen, thereby boosting ECM deposition (Renaud et al., 2020). This leads to scarring, resulting in stiffness, the loss of standard lung architecture, and, ultimately, compromised lung function. Epithelial-mesenchymal transition (EMT) plays a pivotal role in the development of pulmonary fibrosis by contributing to myofibroblast generation. Studies indicate that IGF promotes the pro-fibrotic milieu chiefly through IGF1R signaling pathways, by suppressing matrix metalloproteinases, up-regulating TGFβ, and secreting tissue metalloproteinase inhibitors (Garrett et al., 2019). Further research has shown that TGF-β is crucial for IGF-1 induction in myofibroblasts, and increased levels of IGF-1 in IPF tissues are associated with diminished lung function during disease progression (Hernandez et al., 2020). Mice with bleomycin-induced lung fibrosis and human IPF lung tissue have exhibited elevated IGF-1 levels (Sun et al., 2021). IGF-1 invigorates fibroblast proliferation, protects myofibroblasts from apoptosis, and advocates ECM accumulation, all of which are integral processes in pulmonary fibrosis. Hence, IGF-1 occupies a vital role in the onset and/or proliferation of pulmonary fibrosis.

### 3.3 Connective tissue growth factor (CTGF)

CTGF, or CCN2, is recognized as a prolific instigator of chronic fibrosis hyperplasia (Sgalla et al., 2020a; Effendi and Nagano, 2022). As a cysteine-rich stromal cell protein, it exerts influence over numerous biological processes, including cell proliferation, differentiation, adhesion, angiogenesis, and multiple pathological processes such as tumorigenesis and tissue fibrosis (Ungvari et al., 2017). CTGF is a primary mediator of TGF-β-induced pulmonary fibrosis (Yanagihara et al., 2022). The activation mediated by TGF-β response elements within the CTGF promoter instigates CTGF production, thereby affirming it as a principal arbitrator of TGF-β-induced pulmonary fibrosis (Nguyen et al., 2018). Frequently expressed in mesenchymal cell lines, CTGF often directs tissue regeneration and pathological fibrosis formation via ECM deposition, fibroblast proliferation, and matrix generation (Ramazani et al., 2018). Indeed, the utilization of anti-CTGF antibodies in fibrotic animal models attenuates ECM component expression, enhances survival post-radiation-induced lung injury, and conserves the morphology of alveolar epithelial cells (Bickelhaupt et al., 2017). In IPF patients, CTGF expression is elevated in alveolar cells and mesenchymal fibroblasts (Kasam et al., 2020).

### 3.4 Matrix metalloproteinases (MMPs)

MMPs are proactive contributors to pulmonary fibrosis (Todd et al., 2020). This family of endopeptidases, including MMP-3, MMP-7, and MMP-8, is integral to regulating EMT degradation in IPF (Roque et al., 2020b; Bormann et al., 2022). EMT activation in the lungs is believed to be one of the mechanisms associated with the loss of alveolar cells and the formation of pulmonary fibrosis. These MMPs facilitate pulmonary fibrosis development via several mechanisms: 1. Fostering epithelial-mesenchymal transition (Liu et al., 2020); 2. Inducing m acrophage polarization (Le et al., 2020); 3. Propelling fibroblast migration (Menou et al., 2018); 4. Encouraging abnormal epithelial cell migration and other irregular repair processes (Drankowska et al., 2019). MMP-7 expression is enhanced in both human IPF and mouse fibrosis models (Mahalanobish et al., 2020; Probst et al., 2020). Research has demonstrated that mice with MMP-3 deletion or MMP-7 knockout are safeguarded from bleomycin-induced fibrosis (Mahalanobish et al., 2020). IPF patients exhibit elevated MMP levels in alveolar lavage fluid and blood (Inoue et al., 2020; Espindola et al., 2021). Clinical data suggest an association between elevated MMP-7 levels and an increased risk of mortality and disease progression (Khan et al., 2022).

### 3.5 Exosomes

Exosomes are phospholipid bilayer membranous vesicles, measuring between 30 and 150 nm (Negrete-Garcia et al., 2022). Continuously secreted by a variety of cell types, they transport biologically active substances such as proteins, lipids, and genetic material (DNA, mRNA, miRNAs, and lncRNAs) (Yang et al., 2022). Bronchial epithelial cells primarily generate exosomes in the lungs, which activate fibroblasts, stimulate their differentiation into myofibroblasts, and catalyze excessive extracellular matrix component deposition (Abreu et al., 2021). By delivering miRNA to recipient cells, exosomes regulate diverse inflammatory and angiogenic pathways, playing an instrumental role in inflammation, tissue repair, and fibrogenesis (Rasaei et al., 2022). A multitude of studies underscore the significance of exosomes in IPF pathogenesis, particularly regarding epithelial phenotypes and fibroproliferative responses (d'Alessandro et al., 2021a; Martin-Medina et al., 2018; Parimon et al., 2019). Studies indicate an upregulation of miR-21 in exosomes in mouse pulmonary fibrosis models and human IPF patient serum, correlating with disease progression and mortality (Pastor et al., 2021). A recent study reported an elevated number of Wnt5a-carrying exosomes in IPF patients. These lung fibroblast-derived exosomes exert an autocrine effect, stimulating *in vitro* fibroblast proliferation (Baarsma and Konigshoff, 2017).

**FIGURE 1:**
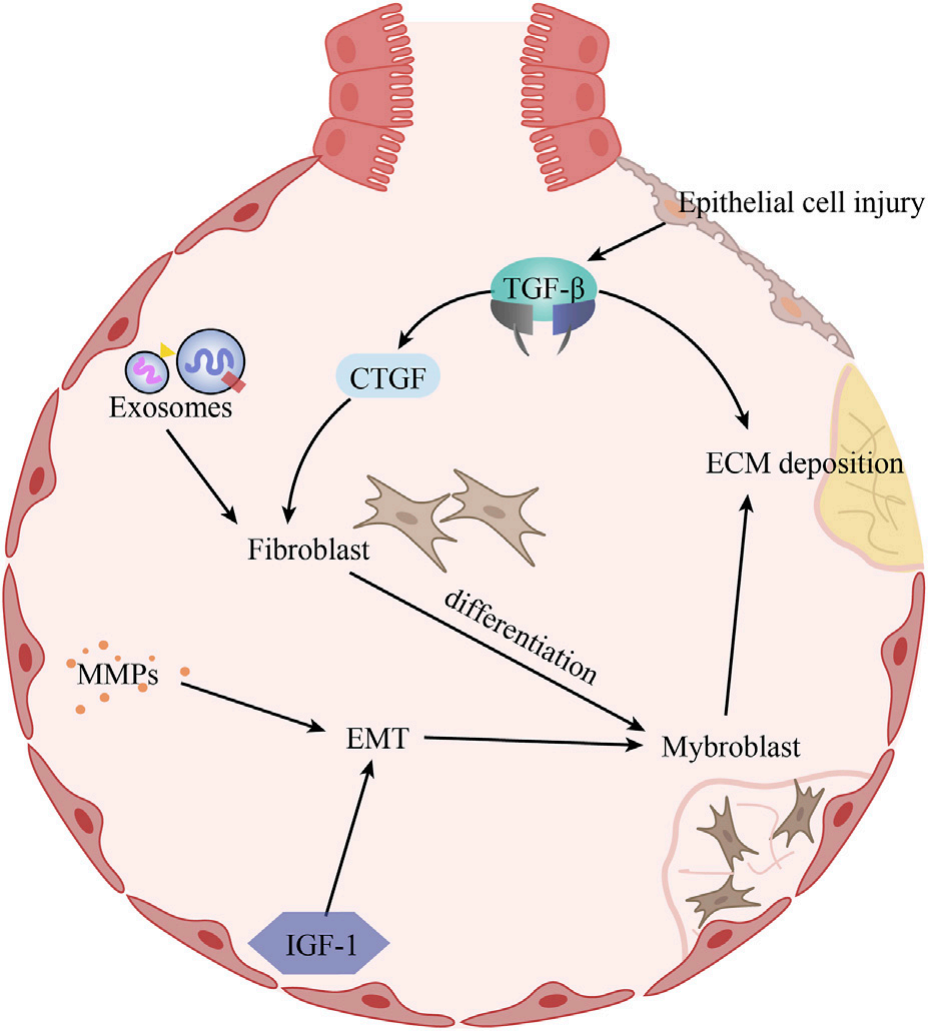
The pathogenesis of idiopathic pulmonary fibrosis. Alveolar epithelial cell injury induces increased TGF-β expression, which promotes ECM deposition and CTGF expression. Under the combined effect of CTGF and exosomes, fibroblasts proliferate and differentiate into myogenic cells. In addition, increased expression of MMPs and IGF-1 in IPF promotes EMT, which in turn affects myogenic cells. The increase in the number of myogenic cells further aggravates ECM deposition.

To summarize, the pathogenesis of idiopathic pulmonary fibrosis is complex, with specific mechanisms appearing to intersect with each other, as outlined in this paper, is depicted in Figure 1.

## 4 Treatment

Currently, conventional medications approved for the clinical treatment of idiopathic pulmonary fibrosis (IPF), such as pirfenidone and nintedanib, only alleviate symptoms without reversing pulmonary fibrosis to facilitate a curative outcome. Consequently, the development of new therapeutic options is imperative. Innovations include investigating novel effects of existing drugs, developing new drugs, and exploring treatments such as stem cell transplantation for IPF. Many drugs are currently under clinical trials, with some advancing to phase 3, thereby expanding the therapeutic arsenal for IPF.

### 4.1 Glucocorticoids and immunosuppressants

Historically, chronic inflammation, seemingly uncontrollable, was perceived as the primary driver of progressive parenchymal fibrosis development (Behr, 2013). Systemic steroids, due to a dearth of effective alternatives, served as the standard treatment for IPF (Raghu et al., 2015). Combinatorial immunosuppressants, including prednisone, azathioprine, cyclophosphamide, and acetylcysteine, were similarly efficacious (Walter et al., 2006). Glucocorticoids and immunosuppressants are usually used for empirical treatment of acute exacerbation of idiopathic pulmonary fibrosis (Naccache et al., 2022). Some studies have shown that the combination of them is beneficial in prolonging the survival of acute patients (Yamazoe and Tomioka, 2018). However, there is currently insufficient evidence to support their routine use. An increasing number of clinical trials have shown that anti-inflammatory therapy and immunosuppressive agents are not effective in conventional treatment of IPF (Ryu et al., 2014; Barratt et al., 2018), leading to the discontinuation of their recommendation for the routine treatment of IPF.

### 4.2 Pirfenidone

Pirfenidone, the inaugural oral antifibrotic drug to receive approval, is a pyridine derivative widely recognized for the treatment of IPF (Vancheri et al., 2018). Its mechanism of action, while multifaceted, remains incompletely understood. Pirfenidone exhibits anti-inflammatory, anti-oxidative, and anti-fibrotic properties, thereby reducing collagen synthesis and deposition in the lungs (Saito et al., 2019). By inhibiting the cytokine TGF-β, it curtails the proliferation, differentiation, and collagen secretion of human lung fibroblasts, decelerates the fibrotic process, and attenuates the decline rate of forced vital capacity (FVC) (Aimo et al., 2022). Several animal model studies in recent years have corroborated the antifibrotic characteristics of pirfenidone (Carrington et al., 2018). It can enhance the prognosis of IPF, reduce mortality, and prolong progression-free survival (Nathan et al., 2017; Vancheri et al., 2018), as has been demonstrated in numerous randomized, placebo-controlled phase III trials (King et al., 2014; Noble et al., 2016). In IPF treatment, pirfenidone demonstrates not only tolerability but also a desirable safety profile (Veit et al., 2019). Therefore, pirfenidone presents a promising treatment avenue for IPF.

### 4.3 Nintedanib

Nintedanib, another approved oral antifibrotic drug, operates as an orally active triple tyrosine kinase receptor inhibitor (Molina-Molina, 2019). Originally conceived as an anti-cancer drug, it later displayed antifibrotic effects and received approval for IPF treatment (d'Alessandro et al., 2021b). Nintedanib effectively impairs the activity of platelet-derived growth factor receptor kinase, fibroblast growth factor receptor kinase, and vascular endothelial growth factor receptor kinase (Hilberg et al., 2008). It suppresses the release of pro-inflammatory and pro-fibrotic mediators, inhibits fibroblast migration and differentiation, and contributes to the blockade of extracellular matrix deposition (Wollin et al., 2015). In bleomycin-induced animal pulmonary fibrosis models, nintedanib exhibited antifibrotic, anti-inflammatory, and vascular remodeling activities (Ackermann et al., 2017). Phase 3 clinical trials demonstrated that, compared to a placebo, nintedanib significantly attenuates the decline rate of forceful lung volume following mild to moderate lung function impairment in IPF patients (Collins and Raghu, 2019). Nintedanib significantly mitigated the risk of disease progression and also demonstrated a mortality-reducing benefit (Richeldi et al., 2014). Moreover, nintedanib exhibited a manageable safety and tolerability profile in clinical trials involving patients (Seibold et al., 2020).

### 4.4 Lung transplantation

Lung transplantation presents itself as the sole treatment alternative that can enhance the quality of life and augment survival rates when previous treatments have failed to yield positive outcomes (Balestro et al., 2019). This life-saving procedure serves as the ultimate solution for advanced stages of IPF. After the diagnosis of IPF, the lung transplantation should be actively evaluated to start the early implantation of transplantation concept. Candidates typically considered for lung transplantation are those with limited treatment alternatives and face a death risk exceeding 50% within 2 years without the transplantation (George et al., 2019). The survival rate post-transplantation has shown consistent improvement over the years, with recent statistics indicating a 1-year survival rate of 88.8% and a 5-year survival rate of 59.2% (Valapour et al., 2021). Despite the continual enhancement in the overall prognosis of lung transplantation, it remains a complex procedure for IPF laden with potential complications. Limitations in the procedure’s application arise from the scarcity of donor organs, the possibility of acute graft-versus-host disease, and the risk of infection (Kapnadak and Raghu, 2021).

### 4.5 Potential therapeutic strategies

#### 4.5.1 Monoclonal antibodies

Pamrevlumab, a humanized monoclonal antibody, targets CTGF, a fibroblast and endothelial cell-secreted glycoprotein pivotal in the pathogenesis of fibrosis (Di Martino et al., 2021). Investigations have demonstrated that pamrevlumab permeates tissues, diminishing effective CTGF levels, which in turn leads to a decline in profibrotic factors, reestablishes the equilibrium between secretion and processing of the extracellular matrix (ECM), and restores tissue homeostasis (Raghu et al., 2016). In a mouse model simulating radiation-induced pulmonary fibrosis, pamrevlumab treatment reversed established lung remodeling and reinstated lung function (Bickelhaupt et al., 2017). Some clinical trials have showcased promising results with pamrevlumab significantly reducing FVC deterioration and slowing disease progression, exhibiting comparable efficacy to pirfenidone and nintedanib (Sgalla et al., 2020a; Richeldi et al., 2020). Data derived from a phase 2 study underscored a favorable safety and tolerability profile for pamrevlumab among the IPF patients participating in the study (Raghu et al., 2016). Pamrevlumab is presently under investigation in a Phase 3 randomized, double-blind, controlled, multicenter trial (Sgalla et al., 2020b). It holds potential as an innovative, safe, and efficacious treatment modality for idiopathic pulmonary fibrosis. Some other monoclonal antibodies, including Atezolizumab, Garadacimab, and Vixarelimab, traditionally employed in the treatment of various other diseases, have also demonstrated therapeutic potential in the clinical management of IPF.

#### 4.5.2 Metformin

Metformin, a time-honored hypoglycemic agent clinically employed in the treatment of type 2 diabetes mellitus, is increasingly recognized for its antifibrotic properties, as corroborated by numerous preclinical investigations (Choi et al., 2016; Rangarajan et al., 2018; Foretz et al., 2019). Metformin was identified to exert pronounced antifibrotic effects by modulating metabolic pathways, impeding transforming growth factor-beta, inhibiting collagen formation, and inducing adipogenic differentiation of lung fibroblasts in IPF patients (Kheirollahi et al., 2019). In the bleomycin-induced pulmonary fibrosis model, metformin mitigated pulmonary fibrosis through the inhibition of TGF-β via the activation of adenosine monophosphate-activated protein kinase. This activation fast-tracked the removal of established fibrosis by promoting myofibroblast inactivation and apoptosis (Choi et al., 2016; Sato et al., 2016). In one particular study, metformin was found to attenuate bleomycin-induced pulmonary fibrosis via the Insulin-like Growth Factor 1 pathway, demonstrating antifibrotic efficacy comparable to that of pirfenidone (Xiao et al., 2020). A retrospective clinical examination of IPF patients with concurrent diabetes revealed that the group treated with metformin exhibited lower all-cause mortality and hospitalization rates compared to the control group (Teague et al., 2022). The robust antifibrotic action of metformin, its low adverse effect profile, and affordability underscore its potential as an antifibrotic therapeutic candidate.

#### 4.5.3 Proton pump inhibitors (PPIs)

PPIs are currently under investigation as potential therapeutic agents for IPF due to the frequent coexistence of gastroesophageal reflux disease and IPF in clinical scenarios (Tran et al., 2021). The antifibrotic potential of PPIs is theorized to stem from their effective inhibition of fibroblast proliferation and downregulation of TGFβ receptor (Ghebremariam et al., 2015). A dose-dependent inhibition of the gene expression of profibrotic markers, such as collagen 1, fibronectin 1, and matrix metalloproteinase 7, by PPIs has also been documented (Nelson et al., 2017). PPIs are further recognized to exhibit antifibrotic effects by upregulating the cytoprotective enzyme heme oxygenase 1 (Ghebre and Raghu, 2016). Anti-fibrotic impacts of PPIs were evident in lung injury models induced by carbon tetrachloride, a liver fibrosis model (Eltahir and Nazmy, 2018). Preclinical *in vivo* studies demonstrated that oral esomeprazole mitigated inflammation and fibrosis in rodent models of bleomycin-induced lung injury, with approximately 50% reduction in each parameter (Ghebremariam et al., 2015). Several studies generally vouch for the beneficial effects of PPI therapy in managing IPF, where PPIs could decelerate lung function deterioration and enhance patient survival (Lee et al., 2011; Lee et al., 2013). Furthermore, a longer duration of PPI use was significantly associated with lower IPF-related mortality in both univariate and multivariate Cox regression analyses (Lee et al., 2016). Given the influence of GERD on IPF progression, proton pump inhibitors are recommended for the management of this disease and have been incorporated into international IPF treatment guidelines (Tran and Suissa, 2021). Lansoprazole, a proton pump inhibitor, is currently undergoing a Phase 3 clinical trial against pulmonary fibrosis, the outcomes of which hold promising prospects.

#### 4.5.4 Stem cell therapy

The exploitation of embryonic stem cells for lung regeneration or repair has gained notable momentum in recent years. Stem cells, essentially immature cells that proliferate and metamorphose into adult cells, demonstrate anti-inflammatory and anti-fibrotic traits, rendering them as a potent potential therapy for fibrotic diseases (Kletukhina et al., 2022). Mesenchymal stem cells (MSCs), pluripotent cells endowed with immunomodulatory and tissue repair capabilities, emerge as a prospective therapeutic avenue for IPF (Yang S. et al., 2022). The potential engagement of MSCs in pulmonary fibrosis hinges on their capacity to generate a plethora of biologically active substances with anti-inflammatory, immunosuppressive, and angiogenic attributes, alongside their ability to minimize extracellular matrix and collagen deposition, thus fostering alveolar repair (Jiang et al., 2015). For instance, MSCs curtail TGF-β1 and tumor necrosis factor-alpha (TNF-α) levels by secreting prostaglandin E2 (PGE2) and hepatocyte growth factor (Dong et al., 2015). Lung spheroid cells (LSCs), forming a distinct spherical structure in culture, comprise stem and support cells native to the lungs that can be reliably cultured from biopsied lung tissue (Surolia et al., 2017). A study revealed that LSC treatment can attenuate and resolve bleomycin-induced fibrosis by reconstructing normal alveolar structure, curtailing collagen accumulation, and myofibroblast proliferation (Henry et al., 2015). When administered intravenously into a mouse model of pulmonary fibrosis, a majority of the cells localized in the animal’s lungs, with the lung spheroid cells demonstrating potent regenerative properties (Dinh et al., 2017). Rats with pulmonary fibrosis treated with spheroid cells manifested healthier lung cells overall and exhibited substantially less lung inflammation and fibrosis (Dinh et al., 2020). In certain clinical studies of IPF, queries regarding the efficacy and safety of stem cell therapy have been addressed. In terms of efficacy, patients receiving the treatment displayed significant improvement in FVC compared to the placebo group (Ntolios et al., 2018; Fishman et al., 2019; Averyanov et al., 2020), with these results being considerably encouraging. No major adverse events were reported, thereby alleviating the majority of concerns (Glassberg et al., 2017; Zhang et al., 2017; Campo et al., 2021). Presently, several Phase I clinical trials of stem cell therapy for IPF are in progress, seeking to fully appraise the safety and feasibility of stem cell therapy. Hence, stem cells’ employment in the treatment of pulmonary fibrosis is deemed a promising therapeutic strategy.

#### 4.5.5 Other potential therapies

Additional therapeutic drugs and methods are undergoing investigation, and although the specific mechanisms remain to be thoroughly scrutinized, it does not impede the advancement of exploratory efforts in IPF treatment strategies. Currently, treprostinil and BI 1015550, which are in Phase 3 clinical trials, are under consideration. Treprostinil is a stable prostacyclin analog, a PGI2 receptor agonist, promoting vasodilation and inhibiting platelet aggregation (Nathan et al., 2022). Certain studies reveal that treprostinil influences cell adhesion and differentiation by inhibiting extracellular regulated kinase signaling, thereby impeding fibroblast proliferation (Blumer et al., 2021). It also demonstrates dose-dependent prevention of fibroblast proliferation to decrease extracellular matrix composition via TGF-beta1 in human peripheral lung fibroblasts (Lambers et al., 2018). BI 1015550, a preferential Phosphodiesterase 4 (PDE4) inhibitor, has been developed for the treatment of IPF and other forms of progressive pulmonary fibrosis (Sgalla et al., 2023). PDE4 inhibition is renowned for its anti-inflammatory and antifibrotic properties (Richeldi et al., 2022). Several experiments have established the inhibition of pulmonary fibrosis by PDE4 inhibitors (Kim et al., 2016; Herrmann et al., 2022). BI 1015550 has been recently evaluated in a phase 2, randomized, double-blind, multi-center, placebo-controlled trial that demonstrated a beneficial treatment effect with an acceptable safety and tolerability profile. Autotaxin, an enzyme involved in lysophosphatidic acid production, is upregulated in IPF patients, thereby constituting a potential target for novel IPF therapeutics. Cudetaxestat is an autotaxin inhibitor, and ARO-MMP7 is an investigational RNA-interfering drug designed to reduce the expression of MMP7 to combat pulmonary fibrosis. Autoantibody Reductive Therapy is mechanistically aimed at ameliorating autoantibody-mediated pulmonary injury.

In addition, smoking patients are advised to quit smoking and given appropriate traditional Chinese medicine adjuvant therapy, which is helpful to improve the quality of life of patients. At the same time, active pulmonary rehabilitation and oxygenotherapy if necessary, which also play a great role in improving the function of the body and stabilizing or slowing down the development of the disease.

**FIGURE 2:**
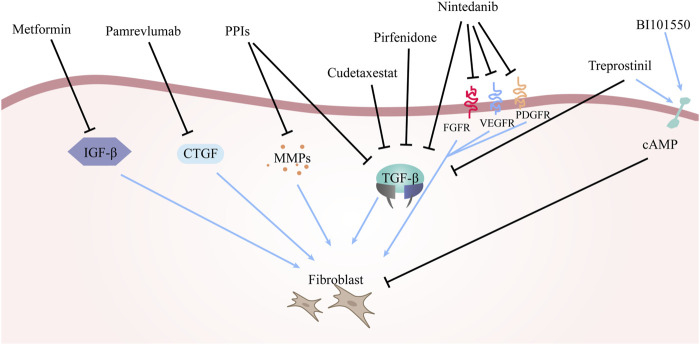
The mechanism of drugs for the treatment of IPF. The arrows in the figure represent the activating effect and the black lines represent the inhibiting effect. The different drugs mentioned above have different principles of action, but the ultimate goal of all drugs is to inhibit fibroblast production and reduce or even block the deposition of ECM.

Various drugs employed to manage idiopathic pulmonary fibrosis are enumerated above, with their mechanisms of action delineated in Figure 2.

While most of these therapies are in their nascent stages of research, they provide substantial reassurance, and it is hoped that they receive expedited approval for the clinical benefit of IPF patients. Research into IPF treatment strategies, buoyed by the introduction of innovative therapeutic agents and treatments, has witnessed a burgeoning number of clinical trials. Some ongoing clinical trials are succinctly presented in Table 1.

**TABLE 1:** A few representative ongoing clinical trials for IPF.

Therapeutic measures	Mechanism	Phase	Status	Trial identifier
Pamrevlumab	CTGF inhibitor	3	Active, not recruiting	NCT03955146
Treprostinil	DP1/EP2 agonist	3	Recruiting	NCT05255991
BI 1015550	PDE4B inhibitor	3	Recruiting	NCT05321069
Lansoprazole	PPIs	3	Recruiting	NCT04965298
Cudetaxestat	Autotaxin inhibitor	2	Active, not recruiting	NCT05373914
Garadacimab	FXIIa mAb	2	Recruiting	NCT05130970
Autoantibody Reductive Therapy	Immunologic Factors	2	Recruiting	NCT03286556
Fipaxalparant	LPA1 antagonist	2	Recruiting	NCT05032066
Orvepitant	NK-1 Receptor Antagonist	2	Recruiting	NCT05185089
Setanaxib	NOX1/4 Inhibitor	2	Recruiting	NCT03865927
Vixarelimab	OSMR-β inhibitor	2	Recruiting	NCT05785624
Bersiporocin	PRS inhibitor	2	Recruiting	NCT05389215
RXC007	ROCK2 inhibitor	2	Recruiting	NCT05570058
Taladegib	Smo inhibitor	2	Recruiting	NCT04968574
TTI-101	STAT3 inhibitor	2	Recruiting	NCT05671835
Bexotegrast	ανβ6 integrin inhibitor	2	Active, not recruiting	NCT04396756
ORIN1001	IRE1a inhibitor	1	Active, not recruiting	NCT04643769
Atezolizumab	PD-L1 inhibitor	1	Recruiting	NCT05515627
ARO-MMP7	RNAi	1	Recruiting	NCT05537025
Human Umbilical Cord MSC	Stem cells	1	Recruiting	NCT05468502
Lung Spheroid Stem Cells	Stem cells	1	Recruiting	NCT04262167
Mesenchymal Stem Cell	Stem cells	1	Recruiting	NCT05016817

## 5 Conclusion

Despite substantial strides in comprehending IPF and formulating innovative treatment strategies, the labyrinthine nature of this disease continues to mandate further exploration. The contemporary perspectives on IPF treatment can be encapsulated as follows.

1 Early Diagnosis and Intervention: The importance of early IPF diagnosis cannot be overstated for administering treatment prior to extensive lung damage. Strategies may encompass heightened awareness and education for healthcare professionals, biomarker utilization, and the creation of avant-garde imaging techniques for early disease detection.
2 Personalized Medicine: As our grasp of the molecular mechanisms underpinning IPF becomes more sophisticated, opportunities may arise to devise personalized treatment strategies aimed at specific pathways or genetic factors in individual patients. This could culminate in more efficacious, customized therapies with diminished side effects.
3 Combination Therapies: Owing to IPF’s multifaceted nature, it is improbable that a single treatment would entirely stymie the disease’s progression. Therefore, combination therapies targeting multiple facets of the disease, including inflammation, fibrosis, and oxidative stress, might enhance IPF management.
4 Regenerative Medicine: Delving into regenerative medicine, inclusive of stem cell therapy and tissue engineering, offers potential for novel IPF treatment strategies. The ultimate aim would be to mend or substitute damaged lung tissue, potentially reversing the disease’s effects.
5 Improved Support and Symptom Management: While a definitive cure for IPF currently eludes us, optimizing symptom management and providing comprehensive support to patients and their families remain paramount. This entails pulmonary rehabilitation, oxygen therapy, and psychological support to assist patients in grappling with the physical and emotional tribulations associated with IPF.
6 Enhanced Collaboration and Research: Sustained collaboration among researchers, clinicians, and pharmaceutical companies is indispensable to catalyze innovation and engender new treatment options for IPF. This calls for a collaborative spirit that encourages data, resource, and knowledge sharing across disciplines to expedite the discovery of new therapeutic targets and augment our understanding of the disease.

In summary, although IPF persists as a formidable and intricate disease, the landscape appears promising with the advent of novel treatment options and research advancements. With an emphasis on early diagnosis, personalized medicine, combination therapies, regenerative medicine, improved support and symptom management, and enhanced collaboration and research, the field stands poised to make substantial progress in the foreseeable future.
